# How far will we need to go to reach HIV-infected people in rural South Africa?

**DOI:** 10.1186/1741-7015-5-16

**Published:** 2007-06-19

**Authors:** David P Wilson, Sally Blower

**Affiliations:** 1 Disease Modeling Group, Semel Institute for Neuroscience and Human Behavior, and UCLA AIDS Institute, School of Medicine, University of California at Los Angeles, 1100 Glendon Avenue, Penthouse 2, Los Angeles, CA 90024, USA; 2 National Centre for HIV Epidemiology and Clinical Research, Faculty of Medicine, University of New South Wales, Level 2, 376 Victoria Street, Sydney, NSW 2010, Australia

## Abstract

**Background:**

The South African Government has outlined detailed plans for antiretroviral (ART) rollout in KwaZulu-Natal Province, but has not created a plan to address treatment accessibility in rural areas in KwaZulu-Natal. Here, we calculate the distance that People Living With HIV/AIDS (PLWHA) in rural areas in KwaZulu-Natal would have to travel to receive ART. Specifically, we address the health policy question 'How far will we need to go to reach PLWHA in rural KwaZulu-Natal?'.

**Methods:**

We developed a model to quantify treatment accessibility in rural areas; the model incorporates heterogeneity in spatial location of HCFs and patient population. We defined treatment accessibility in terms of the number of PLWHA that have access to an HCF. We modeled the treatment-accessibility region (i.e. catchment area) around an HCF by using a two-dimensional function, and assumed that treatment accessibility decreases as distance from an HCF increases. Specifically, we used a distance-discounting measure of ART accessibility based upon a modified form of a two-dimensional gravity-type model. We calculated the effect on treatment accessibility of: (1) distance from an HCF, and (2) the number of HCFs.

**Results:**

In rural areas in KwaZulu-Natal even substantially increasing the size of a small catchment area (e.g. from 1 km to 20 km) around an HCF would have a negligible impact (~2%) on increasing treatment accessibility. The percentage of PLWHA who can receive ART in rural areas in this province could be as low as ~16%. Even if individuals were willing (and able) to travel 50 km to receive ART, only ~50% of those in need would be able to access treatment. Surprisingly, we show that increasing the number of available HCFs for ART distribution ~ threefold does not lead to a threefold increase in treatment accessibility in rural KwaZulu-Natal.

**Conclusion:**

Our results show that many PLWHA in rural KwaZulu-Natal are unlikely to have access to ART, and that the impact of an additional 37 HCFs on treatment accessibility in rural areas would be less substantial than might be expected. There is a great length to go before we will be able to reach many PLWHA in rural areas in South Africa, and specifically in KwaZulu-Natal.

## Background

Accessibility to all types of healthcare is generally inadequate in rural settings of resource-constrained countries [1]. Antiretroviral therapies (ART) are still unattainable for most HIV-infected individuals in these countries, mainly due to the scarcity of drugs [1]. One of the greatest remaining obstacles to receiving treatment in rural areas in these countries is scarcity in health care facilities (HCFs) [1,2]. Considerable discussion has surrounded the many logistical and clinical management difficulties associated with the rollout of ART in Africa [1,3,4]. However, the travel distances required for many people living with HIV/AIDS (PLWHA) in rural areas in Africa to reach an HCF have not yet received much attention. This is the topic of the present work. We address the health policy question 'How far will we need to go to reach PLWHA in rural South Africa?', and specifically, we calculate the distance that PLWHA who live in rural areas in KwaZulu-Natal, South Africa, would have to travel to receive treatment.

Implementing HIV/AIDS treatment programs in rural regions of resource-constrained countries is a realistic goal as several pilot programs have shown [1-5]. In rural Haiti, the HIV Equity Initiative (with Partners in Health [4]) has been extremely effective in introducing and implementing ART [1,2]. The base clinical facility of this program in Cange was set-up in a province with no electricity and limited road access. Trained health workers carry out administration of ART and follow-up despite large distances between the primary health care facility and the many patients needing ART (>5 h by foot for some). Medecins sans Frontieres has assisted in setting up effective clinics in semi-rural regions of South Africa (Lusikisiki in the Eastern Cape [6] and also Khayelitsha in the Western Cape [3]), and similar programs have been successful in Nigeria [1]. UNAIDS has also launched pilot projects to improve ART access in Uganda, Cote d'Ivoire, Chile, and Vietnam [5] (see [7-9] for other recent reviews of the progress in resource-constrained settings).

In rural South Africa there is an overall lack of health care infrastructure, reliable statistics and adequate resources [10]. This greatly hinders the administration and monitoring of ART. The vast majority of PLWHA are referred to tertiary hospitals and then to district hospitals, requiring great travel distances [11]. However, various 'residential' clinics, mobile clinics and community health worker stations have recently been established in some rural regions for basic health and ART care. Most notable is the MSF clinic in Lusikisiki and also a rural, clinic-based antiretroviral drug treatment program piloted in the Mseleni district of northern KwaZulu-Natal [11].

For most PLWHA living in rural areas who do not have local clinic-based ART programs, the maximum distance that they would be able (or willing) to travel for ART is not clear, but it is the primary determinant of health service utilization [12]. Road and public transport networks, geographical barriers, and other factors are also important in determining treatment accessibility. Here, we develop a mathematical model, based upon some simplifying assumptions, that we use to quantify treatment accessibility in rural areas in KwaZulu-Natal in terms of the number of PLWHA that have access to ART.

## Methods

**Figure 1:**
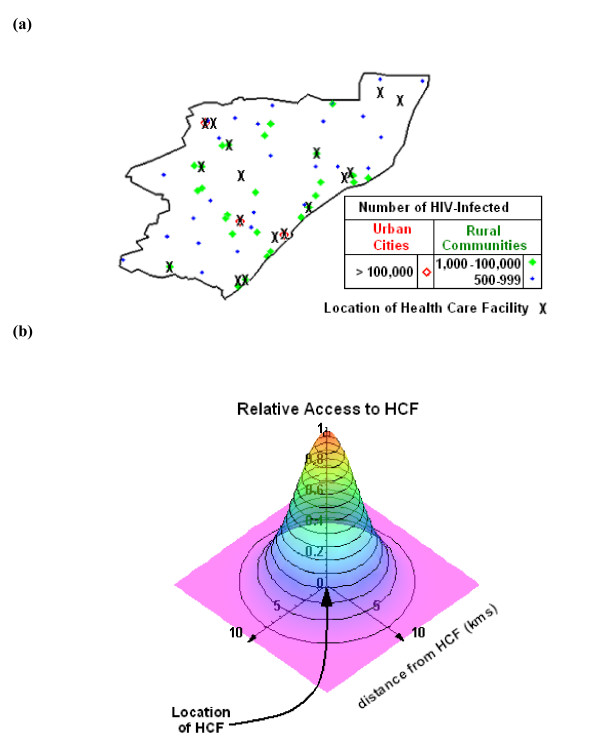
(a) Map of KwaZulu-Natal, indicating (with black crosses) the location of the 17 health care facilities (HCFs) that have been designated for ART rollout by the South African Government, and the spatial distribution of communities distinguished by the number of PLWHA (by both size and color). Durban is the capital city of the province and has more PLWHA than any other community, followed by the cities of Pietermaritzburg and Newcastle; these urban cities are represented by the large red unfilled diamonds. We exclude these three large urban cities and analyze treatment accessibility only for rural communities. (b) Two-dimensional form of a simple function that describes accessibility to ART (assuming that the catchment area has a radius of 10 km). The mathematical form of this accessibility measure is known as a Gaussian, *f*(*d*) = exp (-*kd*^2^). The catchment area radius is defined as the distance where treatment access is reduced to 1% relative to access at a given HCF; the catchment radius is used to calculate the access-scaling parameter *k *in the treatment-accessibility function.

The South African government has outlined detailed operational plans [13] for ART rollout in KwaZulu-Natal that specifies the utilization of 17 currently available HCFs. The spatial location of these 17 HCFs, and the distribution of rural communities with populations of 500–100000 is shown in the map of KwaZulu-Natal in Figure 1a. The 17 HCFs that are specified are a subset of the provincial hospitals of KwaZulu-Natal and do not include community health centers, residential or mobile clinics, or the rest of the centers in the primary health care system. We developed a model to quantify treatment accessibility; the model incorporates heterogeneity in the spatial location of both HCFs and patient population. We modeled the treatment-accessibility region (i.e. the catchment area) around an HCF by using a two-dimensional function (see Figure 1b). We calculated the effect on treatment accessibility of: (1) distance from an HCF (i.e. we vary the radius of the catchment area), and (2) the number of HCFs (the South African Government currently uses 17 HCFs, but at least 54 HCFs are available). We defined treatment accessibility in terms of the number of PLWHA that have access to ART dispensed at an HCF.

To quantify treatment accessibility, we first determined the total number of PLWHA that live in a catchment area of a specific size around each of the HCF (i.e. the 'demand'). We then calculated the 'effective demand' by weighting the 'demand' according to the distance that PLWHA that reside in the catchment area have to travel to reach the HCF. We assumed that accessibility to ART decreases as the distance from the HCF increases; therefore we calculated the 'effective demand' around each HCF by using a distance-discounting measure of ART accessibility based upon a modified form of a gravity-type model [14-16]. Similar functions have been used in infectious disease modeling, specifically to model disease spread [17,18].

To specify our gravity-type model, we used a Gaussian distribution function: *f*(*d*) = exp (-*kd*^2^), where *d *is distance and *k *is an access-scaling parameter that quantifies treatment accessibility. The access-scaling parameter is used to delineate the circumference of the catchment area; '*k*' is defined such that accessibility at the circumference is only 1% of accessibility at the HCF. We used this Gaussian distribution function to calculate the probability that a PLWHA at any given distance from the HCF has access to ART relative to a PLWHA living extremely close to the HCF. The total number of PLWHA in any rural community *i *that has access to any of the (*n*)available HCFs is then calculated by ∑j=1nf(di,j)Ii
 MathType@MTEF@5@5@+=feaafiart1ev1aaatCvAUfKttLearuWrP9MDH5MBPbIqV92AaeXatLxBI9gBaebbnrfifHhDYfgasaacH8akY=wiFfYdH8Gipec8Eeeu0xXdbba9frFj0=OqFfea0dXdd9vqai=hGuQ8kuc9pgc9s8qqaq=dirpe0xb9q8qiLsFr0=vr0=vr0dc8meaabaqaciaacaGaaeqabaqabeGadaaakeaadaaeWbqaaiabdAgaMjabcIcaOiabdsgaKnaaBaaaleaacqWGPbqAcqGGSaalcqWGQbGAaeqaaOGaeiykaKIaemysaK0aaSbaaSqaaiabdMgaPbqabaaabaGaemOAaOMaeyypa0JaeGymaedabaGaemOBa4ganiabggHiLdaaaa@3E63@, where *d*_*i*,*j *_specifies the distance between the rural community *i *and the specified HCF *j*, and *I*_*i *_specifies the number of PLWHA in community *i *(estimated from population levels and published HIV prevalence data of ~9% throughout rural areas [19]).

## Results

**Figure 2:**
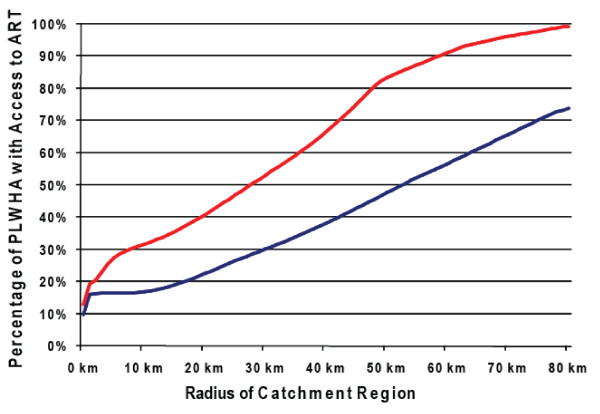
The estimated percentage of PLWHA living in rural areas with access to treatment as a function of the size of the catchment area radius around each HCF. We include the cases of (1) 17 HCFs (blue curve), and (2) 54 HCFs (red curve).

Our calculations show that in rural areas of KwaZulu-Natal, there is a nonlinear relationship between treatment accessibility (i.e. the percentage of PLWHA with access to ART) and the size of the catchment area. This nonlinear relationship is most apparent if 17 HCFS are used, but is also evident if all 54 HCFS are used (Figure 2). In rural areas in KwaZulu-Natal, even substantially increasing the size of a small catchment area (e.g. from 1 km to 20 km) would have a negligible impact on increasing treatment accessibility (i.e. it would only increase by ~2%); whereas increasing the size of a large catchment area could significantly increase treatment accessibility (Figure 2).

Substantially increasing the size of small catchment areas around HCFs in rural areas is unlikely to 'capture' a much larger population of PLWHA, because of the large distances between rural communities. Catchment areas around HCFs in KwaZulu-Natal could be as small as 5 km (Professor Robin Wood, University of Cape Town, personal communication). Thus the percentage of PLWHA who can receive ART in rural areas in this province, if 17 HCFs are used, could be as low as ~16% (~18000/110000) (Figure 2). Even if individuals were willing (and able) to travel 50 km to receive ART, still only ~50% (~52000/110000) of the PLWHA in rural KwaZulu-Natal would be able to access treatment (Figure 2).

The number of HCFs that are utilized (17 versus 54) obviously also affects treatment accessibility (Figure 2). However, surprisingly, our calculations show that increasing the number of available HCFs for ART distribution ~ threefold does not lead to a threefold increase in treatment accessibility in rural KwaZulu-Natal (Figure 2). An increase in treatment accessibility is not proportionate to an increase in HCFs because of HCF locality. Many of the 17 HCFs that are currently utilized for ART distribution are located in or near urban areas, and hence they serve a relatively large number of PLWHA. Many of the additional 37 HCFs that could be utilized are in rural areas and would serve a fairly low number of PLWHA, as rural communities are widely spaced. Therefore, as we have shown, the impact of an additional 37 HCFs on treatment accessibility in rural KwaZulu-Natal would be less substantial than might be expected.

## Discussion and conclusion

Our results show that many PLWHA in rural KwaZulu-Natal are unlikely to have access to ART. We highly recommend that studies collect data on the distance that PLWHA are able (or willing) to travel for treatment. This will commence the facilitation of discussion and decisions on ART allocation, and would help focus goals towards enabling PLWHA to access ART. A mobile clinic that travels between remote communities to take healthcare workers and resources to locations of demand in Nigeria is an initiative that other regions could implement. If new HCFs were to be constructed to increase ART access and other basic health care needs, optimization techniques could be used to determine the most appropriate location [20]. Following initial consultation and drug disbursement, frequent monitoring must also be sustained long-term. Given the limited experience with large-scale ART programs in rural resource-constrained countries, learning from newly implemented programs is essential in informing the direction and priorities that will ensure long-term sustainability, quality, and success.

Although there is no single solution regarding how best to introduce ART into resource-constrained settings, obviously more drugs, healthcare personnel and HCFs are needed in these countries [1,3], particularly in rural areas. Innovative programs are urgently needed to remove the substantial barriers for PLWHA in rural regions (such as long travel distances, shortage of trained health professionals, lack of transportation and community stigma toward PLWHA). Addressing the critical need for adequate care for PLWHA in rural areas requires immediate investment in rural areas in basic infrastructure, particularly human resources and rural primary health facilities. Our results have shown that there is a great length to go before we will be able to reach many PLWHA in rural areas in Africa, and specifically in KwaZulu-Natal.

## Competing interests

The author(s) declare that they have no competing interests.

## Authors' contributions

DPW and SMB developed the concept and study design, and wrote and edited the manuscript. DPW conducted mathematical analyses. SMB also supervised the project.

## Pre-publication history

The pre-publication history for this paper can be accessed here:


